# Unraveling the role of type 1 fimbriae in *Salmonella* pathogenesis: insights from a comparative analysis of *Salmonella* Enteritidis and *Salmonella* Gallinarum

**DOI:** 10.1016/j.psj.2023.102833

**Published:** 2023-06-03

**Authors:** Agata Mikolajczyk-Martinez, Maciej Ugorski

**Affiliations:** Department of Biochemistry and Molecular Biology, Faculty of Veterinary Medicine, Wroclaw University of Environmental and Life Sciences, Wroclaw, Poland

**Keywords:** *Salmonella* Enteritidis, *Salmonella* Gallinarum, type one fimbriae, pathogenesis, chicken cell line

## Abstract

Significant differences in pathogenicity between *Salmonella* Enteritidis and *Salmonella* Gallinarum exist despite the fact that *S*. Gallinarum is a direct descendant of *S*. Enteritidis. It was hypothesized that such various properties may be in part the result of differences in structure and functions of type 1 fimbriae (**T1Fs**). In *S.* Enteritidis, T1Fs bind to oligomannosidic structures carried by host cell glycoproteins and are called mannose-sensitive T1Fs (**MST1F**). In *S.* Gallinarum, T1Fs lost ability to bind such carbohydrate chains, and were named mannose-resistant MRT1Fs (**MRT1F**). Therefore, the present study was undertaken to evaluate the role of MST1Fs and MRT1Fs in the adhesion, invasion, intracellular survival and cytotoxicity of *S*. Enteritidis and *S*. Gallinarum toward chicken intestinal CHIC8-E11cells and macrophage-like HD11 cells. Using mutant strains: *S.* Enteritidis fimH::kan and *S.* Gallinarum fimH::kan devoid of T1Fs and in vitro assays the following observations were made. MST1Fs have a significant impact on the chicken cell invasion by *S.* Enteritidis as MST1F-mediated adhesion facilitates direct and stable contact of bacteria with host cells, in contrast to MRT1Fs expressed by *S*. Gallinarum. MST1Fs as well as MRT1Fs did not affected intracellular viability of *S*. Enteritidis and *S*. Gallinarum. However, absolute numbers of intracellular viable wild-type *S*. Enteritidis were significantly higher than *S.* Enteritidis fimH::kan mutant and wild-type *S*. Gallinarum and *S.* Gallinarum fimH::kan mutant. These differences, reflecting the numbers of adherent and invading bacteria, underline the importance of MST1Fs in the pathogenicity of *S*. Enteritidis infections. The cytotoxicity of wild-type *S.* Enteritidis and its mutant devoid of MST1Fs to HD11 cells was essentially the same, despite the fact that the number of viable intracellular bacteria was significantly lower in the mutated strain. Using HD11 cells with similar number of intracellular wild-type *S*. Enteritidis and *S.* Enteritidis fimH::kan mutant, it was found that the lack of MST1Fs did not affect directly the cytotoxicity, suggesting that the increase in cytotoxicity of *S*. Enteritidis devoid of MST1Fs may be associated with crosstalk between T1Fs and other virulence factors.

## INTRODUCTION

Since the first European Food Safety Authority report about zoonotic diseases was released (2004), *Salmonella enterica* spp. *enterica* has become the second most common cause of foodborne zoonosis in humans (Plym and Wierup, 2006; EFSA and ECDC, 2021a,b). Poultry is the main source of salmonellosis in humans, resulting from the consumption of contaminated meat, eggs and egg products (Chlebicz and Śliżewska, 2018; EFSA and ECDC, 2021b). *Salmonella* serovar, which most frequently causes such infections in Europe, is *Salmonella* Enteritidis (***S.* Enteritidis**) (Authority and European Centre for Disease Prevention and Control, 2014, European Food Safety Authority and European Centre for Disease, 2017, 2021b). Infections caused by *S.* Enteritidis are usually characterized by lack of obvious clinical symptoms and typically are limited to gastroenteritis and diarrhea. Only occasionally, in individuals with immunological deficiencies, infections take the form of systemic disease that can lead to death (Kogut et al., 1994; Milczarek et al., 2019; Abebe et al., 2020). This serovar, infecting both humans and poultry, is referred to as host unrestricted (Bäumler et al., 1998; Uzzau et al., 2000). Another *Salmonella* serovar common in poultry is *S.* Gallinarum biovar Gallinarum (***S*. Gallinarum**), which does not pose an epidemiological threat to humans, but is a serious economic problem in low- and middle-income countries (Sun et al., 2021; Vaid et al., 2021; Zhang et al., 2021). *S*. Gallinarum causes systemic infections called fowl typhoid, with characteristic clinical symptoms and a very high mortality rate of infected chickens (Shivaprasad, 2000). Unlike *S.* Enteritidis, *S.* Gallinarum can infect only poultry and therefore represents *Salmonella* serovars that are referred to as host specific (Uzzau et al., 2000).

Differences in the course of the disease reflect of differences in the pathogenic mechanisms of *S*. Enteritidis and *S*. Gallinarum infections, which include such steps as adhesion to and invasion of various host cells, intracellular survival and cytotoxicity toward host cells. It was found that *S*. Enteritidis and other host unrestricted serovars such as Typhimurium, Hadar, and Infantis invaded chicken kidney epithelial (**CKC**) and lung cells, chicken hepatoma leghorn male hepatoma cells, chicken fibroblastic-like DF-1 and macrophage-like HD11 cells in significantly higher numbers than *S*. Gallinarum and *S*. Pullorum (Setta et al., 2012; Rossignol et al., 2014), resulting in better intracellular bacterial survival (Setta et al., 2012). Confirming these findings, it was shown that *S*. Typhimurium and *S*. Dublin had stronger invasion abilities into chicken primary macrophages and HD11 cells than *S*. Gallinarum. However, a higher survival rate was observed for this latter serovar, despite the fact that *S*. Gallinarum showed poorer net replication (Huang et al., 2019; Huang et al., 2020). In contrast, similar invasion and survival rates within chicken HD11 macrophages were observed for *S*. Galliarum and *S*. Typhimurium, *S*. Dublin, and *S*. Choleraesuis by Chadfield et al. (2003). Avian host-specific *S*. Gallinarum was also characterized by significantly lower cytotoxicity against chicken primary macrophages and HD11, than host unrestricted *S*. Typhimurium and *S*. Dublin (Huang et al., 2019; Huang et al., 2020).

Even though *S*. Gallinarum is a direct descendant of *S*. Enteritidis with 99.7% homology of their orthological genes (Thomson et al., 2008), significant differences in pathogenicity between *S*. Enteritidis and *S*. Gallinarum exist. Therefore, it was proposed that such various properties may be the result of differences in just a few specific virulence factors expressed by these 2 serovars (Wigley et al., 2002; Thomson et al., 2008). For example, genome comparison of *S*. Gallinarum and *S*. Enteritidis revealed the presence of specific point mutations in several genes: *sipA, sopE, sopD*, and *sopA* coding main effector proteins of type III secretion system (**T3SS**) encoded by *Salmonella* pathogenicity island 1 (**SP-1**) (**T3SS-1**), which seems responsible for the low invasion of *S*. Gallinarum into host cells (Rossignol et al., 2014). Another important *Salmonella* virulence factor is type 1 fimbriae (**T1Fs**) (Ugorski et al., 2011; Kolenda et al., 2019), which bind *S.* Enteritidis to oligomannosidic structures carried by many eukaryotic membrane-bound and secreted glycoproteins and therefore are called mannose-sensitive type 1 fimbriae (**MST1F**) (Kisiela et al., 2006). In contrast, T1Fs expressed by *S.* Gallinarum lost their ability to bind such carbohydrate chains, and therefore were named mannose-resistant type 1 fimbriae (**MRT1F**) (Chadfield et al., 2003; Kisiela et al., 2005; Barrow and Neto, 2011; Rossignol et al., 2014). In poultry, it was found that MST1Fs were involved in the adhesion of *S*. Typhimurium to immobilized mucus and enterocytes isolated from chicken ceca and the small intestine and colonization of broiler ceca (Oyofo et al., 1989; Craven et al., 1992). Also, MST1Fs enhanced cecal and oviduct colonization by *S*. Enteritidis in laying hens (Thiagarajan et al., 1996; De Buck et al., 2004), mediating adhesion of bacteria to chicken tubular gland cells and secretions of the isthmus (De Buck et al., 2003). However, others have shown that MST1Fs are not important in the adherence of *S*. Enteritidis to gut explants and cecal colonization of 1-day-old chicks (Allen-Vercoe and Woodward, 1999a,b). During the extraintestinal phase of infections and systemic salmonellosis the entrance of *Salmonella* into host macrophages is a key and necessary step in colonization by bacteria of vital internal organs such as liver and spleen (Haraga et al., 2008; Fàbrega and Vila, 2013). On the cellular level, the infection of macrophages by *Salmonella* includes such processes as invasion and ability to survive inside host cells (Fields et al., 1986; Vazquez-Torres et al., 1999). According to Rajashekara et al. (2000), MST1Fs do not participate in the invasion of chicken macrophage HD-11 and MQ-NCSU cell lines by *S*. Enteritdis. Confirming this data, it was found that MST1Fs are not involved in colonization of spleen and liver in 5-day-old chickens and laying hens (Thiagarajan et al., 1996; Rajashekara et al., 2000). On the other hand, in a 1-day-old chickens, MST1F-negative mutant *S*. Enteritidis had a lower ability than wild type *S*. Enteritidis to colonize liver and spleen (Dibb-Fuller and Woodward, 2000). Unlike MST1F, MRT1F expressed by *S*. Gallinarum play no role in adhesion to epithelial cells (Ugorski et al., 2011; Kolenda et al., 2019). However, there were some indications that MRT1Fs may be involved in binding *S*. Gallinarum to chicken leukocytes (Guo et al., 2009; Kuźmińska-Bajor et al., 2012).

Taking into account conflicting data on the role of MST1Fs in the pathogenicity of *S*. Enteritidis and limited data on the involvement of MST1Fs and MRT1Fs in all consecutive steps of *S*. Enteritidis and *S*. Gallinarum infections in poultry, the present study was undertaken to evaluate the role of MST1Fs and MRT1Fs in the adhesion, invasion, intracellular survival and cytotoxicity of, respectively, *S*. Enteritidis and *S*. Gallinarum using chicken intestinal CHIC8-E11cells and macrophage-like HD11 cells.

## MATERIALS AND METHODS

### Bacterial Strains and Culture Conditions

The *S.* Enteritidis strain (isolate no. 327) was recovered from a broiler chicken and *S.* Gallinarum strain (isolate no. 589/02) was collected from a laying hen with clinical signs of fowl typhoid (Kisiela et al., 2005; Kisiela et al., 2006). For all assays, bacteria were grown in Luria Broth (**LB**) medium at 37°C, under stationary conditions, and passaged 3 times for optimal T1F expression.

### Flow Cytometry

The presence of T1Fs was analyzed by flow cytometry as previously described (Kisiela et al., 2006). Briefly, bacteria suspended in phosphate-buffered saline (**PBS**) were incubated with an equal volume of 4% paraformaldehyde (Pol-Aura, Morag, Poland) for 20 min at room temperature (**RT**). After washing with PBS, to block nonspecific binding, bacteria were resuspended in 1% bovine serum albumin (**BSA**) (BioShop, Mainway Burlington, Ontario, Canada) and incubated for 30 min at RT. For detection of T1Fs, *Salmonella* were incubated with rabbit polyclonal anti-FimH antibodies (Kisiela et al., 2005) and FITC-conjugated goat anti-rabbit IgG (Merck KGaA, Darmstadt, Germany). Flow cytometry analysis was performed using BD FACS Lyric and Flow Jo v10 software (Becton-Dickinson, Warsaw, Poland)**.** For each sample, the fluorescence of 10,000 events was measured.

### Mutant Construction and Phenotypic Analysis

*S.* Enteritidis and *S.* Gallinarum mutants with disrupted *fimH* gene, and therefore devoid of T1Fs, were obtained as described previously (Datsenko and Wanner, 2000; Kuźmińska-Bajor et al., 2012) with minor modifications. Briefly, bacteria, carrying pKD46 plasmid coding recombinase genes (kindly provided by The Coli Genetic Stock Center, Yale University, USA) were subjected to homologous recombination with linear DNA cassette containing the *kan^R^* gene and homologous sequences to the *fimH* gene. The *kan^R^* cassette was amplified by PCR using pKD4 plasmid (kindly provided by The Coli Genetic Stock Center, Yale University, USA) as a template and a pair of primers: O1901fimHdelfwd and O1902fimHdelrev (Zeiner et al., 2012). Mutant strains were verified by sequencing relevant PCR amplicons. The resulting mutants were named *S.* Enteritidis fimH::kan and *S.* Gallinarum fimH::kan. The growth rate of all strains was assessed by optical density measurement at 600 nm every half an hour for 12 h, in an automatic microplate reader (Tecan, Basel, Switzerland). The morphology of all strains was analyzed by fluorescent microscopy (Opta-Tech, Warsaw, Poland) after staining bacteria with 0.05 mg/mL acridine orange (Merck KGaA, Darmstadt, Germany).

### Cells and Cell Culture

The chicken intestinal epithelial CHIC-8E11 cells (Ali et al., 2020) were kindly provided by Prof. Peter Schierack (Brandenburg University of Technology Cottbus-Senftenberg, Faculty of Environment and Natural Sciences) and chicken macrophage-like HD11 cells (Beug et al., 1979) were kind gift of Dr Clare Pridans (Roslin Institute, Easter Bush, Midlothian, Scotland, UK). CHIC-8E11 cells were cultured in DMEM F12 medium (Biowest, Nuaille, France) supplemented with 5% heat-inactivated fetal bovine serum (**FBS**, Thermo Fisher Scientific, Waltham, MA), 2 mmol/L L-glutamine, 100 U/mL penicillin and 100 μg/mL streptomycin (all purchased from Sigma-Aldrich; Merck KGaA, Darmstadt, Germany) (complete DMEM F12). HD11 cells were grown in RPMI 1640 medium (Biowest, Nuaille, France) supplemented with 10% FBS L-glutamine and antibiotics as above (complete RPMI 1640).

For in vitro assays, HD11 cells were activated with 100 ng/mL of phorbol myristate acetate (**PMA**) or 1 µg/mL of lipopolysaccharide (**LPS**) (both purchased from Merck KGaA, Darmstadt, Germany) 24 h before infection with *Salmonella*. Stimulation of monocyte and macrophage-like cell lines is a common procedure while working with these types of cells. It is well known that PMA-stimulated cells have a phenotype of M0 macrophages (Genin et al., 2015; Lund et al., 2016) and similarly to unstimulated cells do not display pro-inflammation phenotype**.** In order to gain pro-inflammatory phenotype, monocytes were incubated with LPS (Ciesielska et al., 2021; Genin et al., 2015)**.**

### Adherence/Invasion/Survival Assays

For the adherence assay, intestinal epithelial CHIC-8E11 cells (1.7 × 10^5^/well) were seeded into 24-well tissue culture plates (Cellstar, Greiner Bio-One GmbH, Kremsmunster, Austria) 72 h before *Salmonella* infection. Macrophage-like HD11 cells (2.5 × 10^5^/well) were seeded 24 h before bacterial infections in the absence or presence of PMA or LPS. Bacterial suspensions were added at a Multiplicity of Infection (**MOI**) 1:50, unless otherwise indicated, and incubated at RT for 1 h. Thereafter, nonadherent bacteria were removed by washing the wells with PBS 3 times. Suspensions of extracellular and intracellular bacteria, released after lysis of eukaryotic cells with 0.5% Triton X-100 (Merck KGaA, Darmstadt, Germany) for 20 min, were serially diluted with PBS, plated on LB-agar, and incubated overnight at 37°C. The next day, bacterial colonies were counted to calculate the colony forming units (**CFU**).

For the invasion assay, bacterial suspensions were added at a MOI 1:50 (unless otherwise indicated). After 1 h-incubation at RT and washing out nonadherent cells with PBS, CHIC-8E11 and HD11 cells were grown for another 0.5 h at RT in the presence of 100 µg/mL gentamicin (Merck KGaA, Darmstadt, Germany) to kill the adherent bacteria (gentamicin protection assay). Then the cells were washed as above. To calculate the number of CFU, suspensions of intracellular bacteria, released after lysis of eukaryotic cells with 0.5% Triton X-100 were treated as above. The adherence and invasion assays were also performed in the presence of 0.2 M D-mannose.

For the survival assay, bacteria were incubated first for 1 h and then for 0.5 h with CHIC-8E11 and HD11 cells as described for the invasive assay, and then for an additional 23.5 h in complete RPMI 1640 containing 10 µg/mL gentamicin. After washing with PBS and detachment with EDTA-trypsin (0.05%/ 0.02%), viable eukaryotic cells were counted using the trypan blue exclusion assay (Strober, 2001). Amounts of intracellular viable bacteria were determined as described for the invasive assay. Taking into account that *S.* Enteritidis and *S*. Gallinarum differ with respect to their invasion and cytotoxicity against host cells, intracellular bacterial survival was expressed as a fold change of live bacteria using the following formula:

$$\text{Fold change}=\text{CF}U_{24h}/100\;\text{viable eukaryotic cells after}\;24\;h/\text{CF}U_{1.5h}/100\;\text{viable eukaryotic cells after}1\;h$$

### Cytotoxicity Assay

*Salmonella*-mediated cytotoxicity was determined by measuring the activity of released lactate dehydrogenase (**LDH**) into the culture supernatant from eukaryotic cells infected with bacteria at 24 h postinfection, using the CyQuant LDH Cytotoxicity Assay (ThermoFisher Scientific, Waltham, MA). Briefly, CHIC-8E11 and HD11 cells were grown in 24-well tissue culture plates (Cellstar, Greiner Bio-One GmbH, Kremsmunster, Austria) and infected with bacteria as described for the survival assay. LDH assay was performed according to the manufacture's protocol. Absorbance was measured at 490 nm at a microplate reader (Tecan, Basel, Switzerland). The percentage of cytotoxicity was calculated using the following formula:

%cytotoxicity=(Salmonella−mediated LDH activity−spontaneous LDH activity)÷(maximum LDH activity−spontaneous LDH activity)×100%

where *Salmonella*-mediated LDH activity correspond to the activity of LDH released by infected cells, spontaneous LDH activity correspond to the activity of LDH released from noninfected cells, and maximum LDH activity corresponded to the activity of LDH released from noninfected cells treated with lysis buffer supplied by the manufacturer.

### Caspase-1, -3, and -7 Activity Assays

In order to measure caspase-1, -3, and -7 activities, FLICA 660 caspase-1 and FLICA 660 caspase-3/7 fluorometric assay (Immunochemistry Technologies, Davis, CA) was performed according to the manufacturer's protocol. HD11 cells (8 × 10^5^/well) were seeded in 6-well tissue culture plates (Cellstar, Greiner Bio-One GmbH, Kremsmunster, Austria) 24 h before infection. Cells were infected with bacteria at an MOI 1:25. After 6 h and 24 h postinfection, cells were washed 3 times with PBS, detached by EDTA-trypsin, washed again with PBS, and incubated with APC-YVAD-FMK peptide to determine the activity of caspase-1 or APC-DEVD-FMK peptide to determine the activity of caspase-3 for 30 min at 37°C. Dead cells were detected with NucGreen Dead 488 reagent (Thermo Fisher Scientific) after incubation for another 30 min. The cells were then washed and subjected to FACS analysis. Fluorescence was measured in FL-1 on the BD FACS Lyric (Becton Dickinson). A total of 10,000 events were recorded. Data were processed and analyzed using Flow Jo v10 software.

### Statistical Analysis

All statistical calculations were performed in GraphPad Prism (GraphPad Software Inc., La Jolla, CA). One-way ANOVA test with Tukey's multiple comparison post hoc test were performed. A p-value of less than 0.05 was considered statistically significant. Data are presented as the means standard deviation (**SD**) or standard error of the mean (**SEM**). *P < 0.05; **P < 0.01; ***P < 0.005; ****P < 0.0001. Assays were conducted in triplicate and were independently repeated at least 3 times.

## RESULTS

### Characterization of *S.* Enteritidis and *S.* Gallinarum *fimH* Gene Mutants Devoid of T1Fs

**Figure 1:**
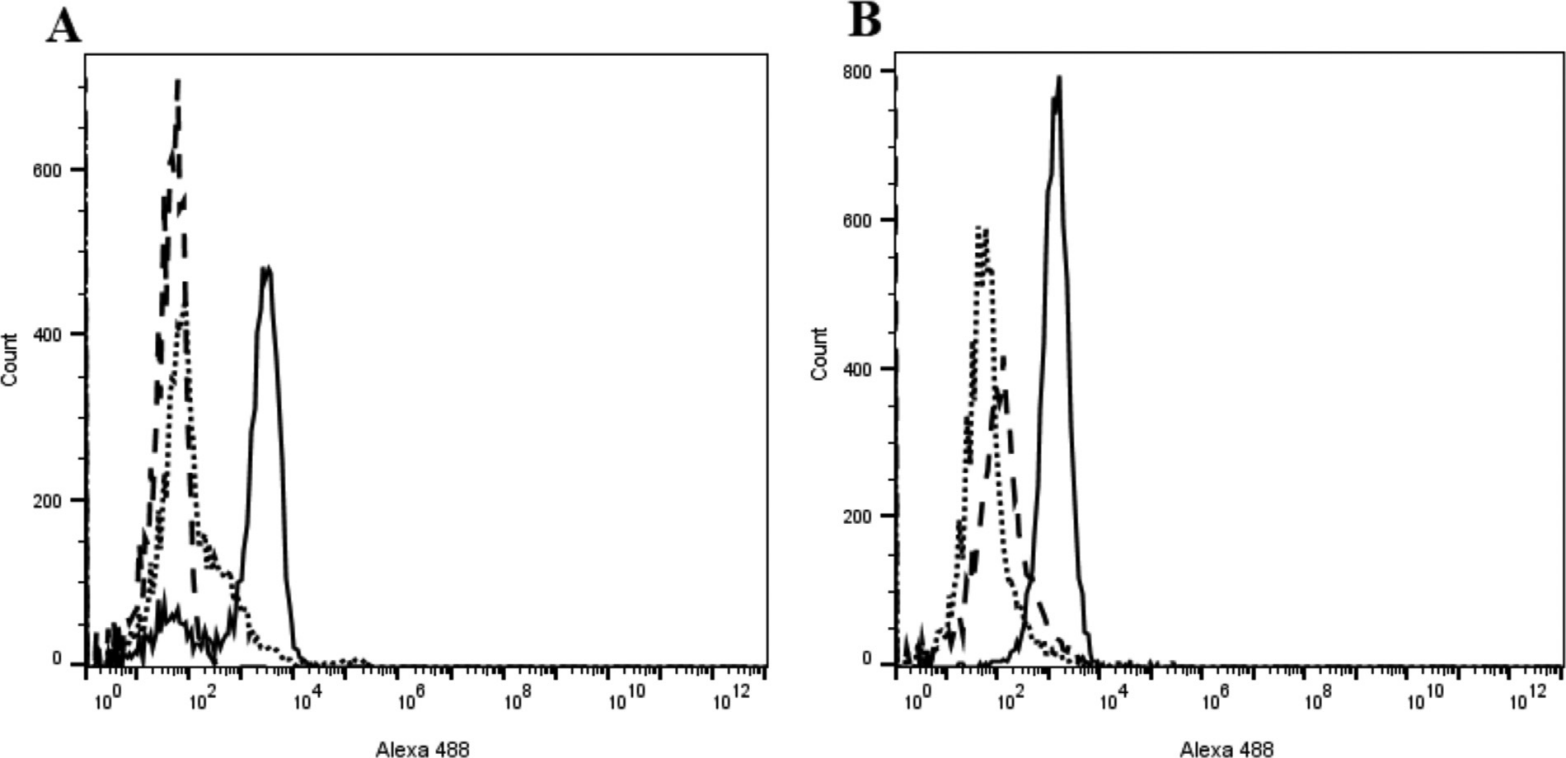
Flow cytometric analysis of FimH expression on (A) wild-type *S.* Enteritidis and its mutant devoid of T1Fs (*S.* Enteritidis fimH::kan) and (B) wild-type *S.* Gallinarum and its mutant devoid of T1Fs (*S.* Gallinarum fimH::kan). Solid lines show the binding of rabbit polyclonal anti-FimH antibodies to wild-type *Salmonella* serovars. Dashed lines show the binding of rabbit polyclonal anti-FimH antibodies to mutants devoid of FimH protein. Dotted lines show the nonspecific binding of the secondary goat antibodies directed against rabbit immunoglobulins. The percentage of cells with fluorescence intensities that lie within the gated area is shown in the top righthand corner of each histogram.

The flow cytometry analysis of bacteria stained with anti-FimH antibody showed that wild-type *S.* Enteritidis and *S.* Gallinarum strains express T1Fs at similar levels, and that the *S.* Enteritidis fimH::kan and *S.* Gallinarum fimH::kan mutants do not elaborate T1Fs (Figure 1). The mutant strains of *S.* Enteritidis and *S.* Gallinarum with knock-out of the *fimH* gene did not differ in morphology and in vitro growth rate from wild-type bacteria.

### Interaction of Wild-Type *S.* Enteritidis and *S.* Gallinarum and Their Mutants Devoid of T1Fs With Chicken Intestinal Epithelial and Macrophage-Like Cell Lines

**Figure 2:**
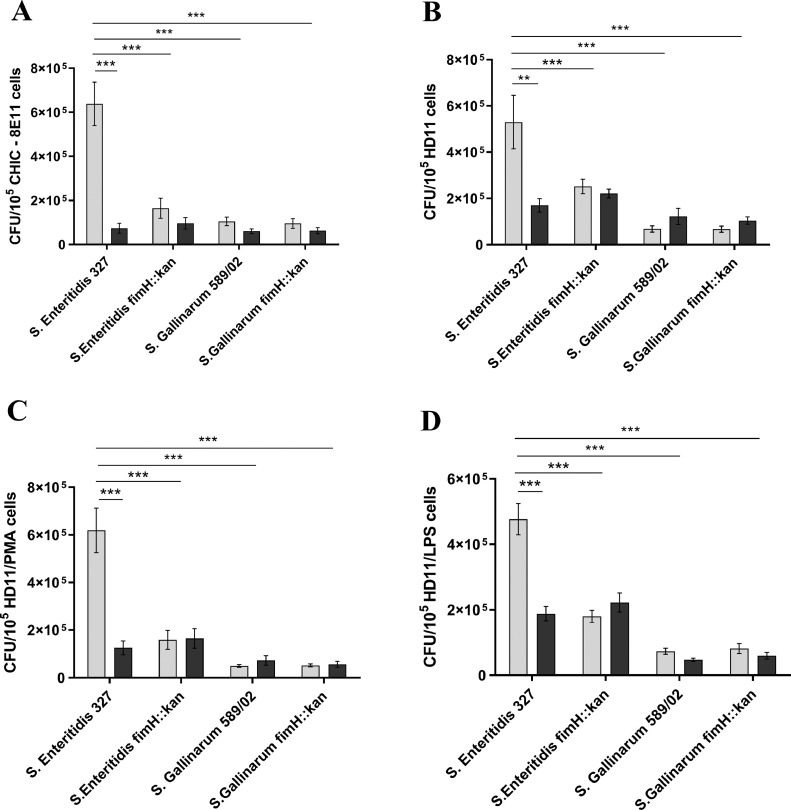
Adherence of wild-type *S.* Enteritidis and *S.* Gallinarum and their mutants, respectively, *S.* Enteritidis fimH::kan and *S.* Gallinarum fimH::kan devoid of T1Fs to (A) chicken intestinal epithelial CHIC-8E11 cells, (B) nonactivated chicken macrophage-like HD11 cells, (C) PMA-activated HD11 cells, and (D) LPS-activated HD11 cells. The numbers of adherent bacteria were expressed as colony forming units (CFU). The results are the means ± SEM of 3 independent biological replicates, each consisting of 3 technical replicates (*n* = 9). *P < 0.05; **P < 0.01; ***P < 0. 005. In order to determine the statistical significance, an analysis of variance with the one-way ANOVA test was performed, and then the multiple comparison procedure was performed with the Tukey's test. Light gray bars represent the binding of bacteria to cells in the absence of 0.2 M D-mannose; the dark gray bars represent the binding of bacteria to cells in the presence of 0.2 M D-mannose.

The adhesion assay revealed that wild-type *S.* Enteritidis bound to chicken epithelial CHIC-8E11 cells and macrophage-like HD11 cells untreated and treated with PMA or LPS in much higher numbers (about 6, 7, 6, and 12 times better, respectively) than the wild-type *S.* Gallinarum strain (Figure 2). Both serovars adhered to intestinal epithelial cells and macrophages in similar numbers. As expected, the adhesion of mutant *S.* Enteritidis fimH::kan strain devoid of MST1Fs to CHIC-8E11 cells and HD11 cells untreated and treated with PMA or LPS was highly diminished (about 4, 2, 3, and 4 times, respectively) in comparison to wild-type bacteria. The binding of *S.* Enteritidis fimH::kan mutant was similar to adhesion of wild-type *S.* Enteritidis in the presence of 0.2 M D-mannose. On the other hand, *S.* Gallinarum fimH::kan mutant bound to CHIC-8E11 and HD11 cells to the same extend as wild-type *S.* Gallinarum.

**Figure 3:**
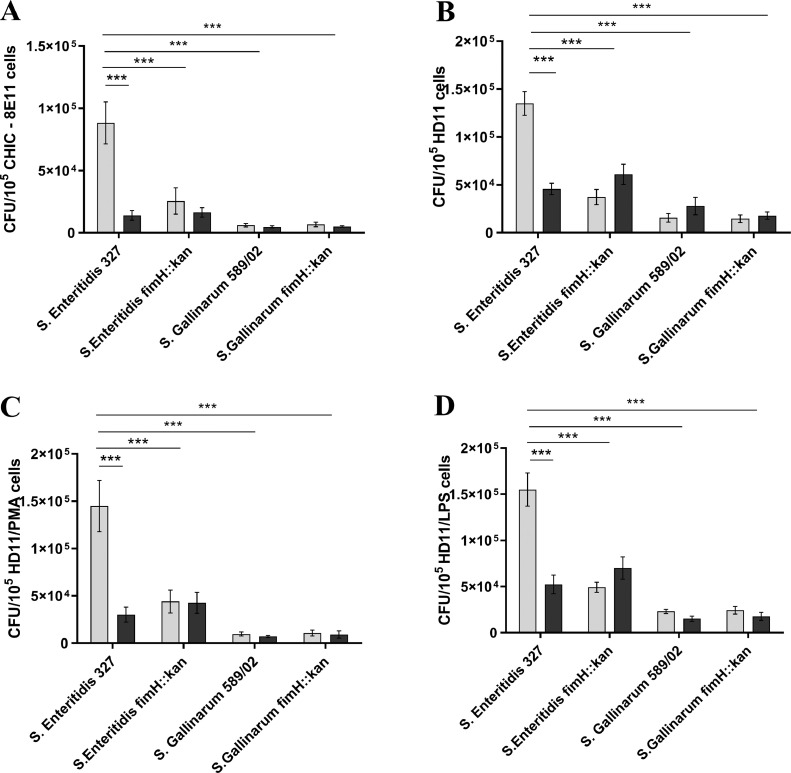
Invasion of chicken intestinal epithelial CHIC-8E11 cells (A), chicken macrophage-like cells HD11 (B), macrophage-like HD11 cells treated with PMA (C) or LPS (D) by wild-type *S*. Enteritidis and *S.* Gallinarum and their *S.* Enteritidis fimH::kan and *S.* Gallinarum fimH::kan mutants devoid of T1Fs. The numbers of invaded bacteria were expressed as colony forming units (CFU). The results presented are the means ± SEM of 3 independent biological replicates, each of which included 3 technical replicates (*n* = 9). *P < 0.05; **P < 0.01; ***P < 0. 005. In order to determine the statistical significance, an analysis of variance with the one-way ANOVA test was performed, and then the multiple comparison procedure was performed with the Tukey's test. Light gray bars show bacterial binding to cells in the absence of 0.2 M D-mannose; the dark gray bars show the binding of bacteria to cells in the presence of 0.2 M D-mannose.

Wild-type *S.* Enteritidis invaded CHIC-8E11 cells and HD11 cells untreated and treated with PMA or LPS (about 15, 8, 7, and 15 times, respectively) better than wild-type *S.* Gallinarum strain (Figure 3). No differences in the number of intracellular bacteria were found between chicken epithelial cells and macrophages. The invasion by *S.* Enteritidis was highly dependent on the expression of MST1Fs, as the number of intracellular bacteria was about 3.5 times lower than the *S.* Enteritidis fimH::kan strain lacking MST1Fs. The invasion of *S.* Enteritidis fimH::kan mutant was similar to the invasion of wild-type *S.* Enteritidis in the presence of 0.2 M D-mannose. In contrast, *S.* Gallinarum fimH::kan mutant invaded chicken cells similarly to wild-type *S.* Gallinarum.

### Survival of Wild-Type *S.* Enteritidis and *S.* Gallinarum and Their Mutants Devoid of T1Fs in Chicken Intestinal Epithelial and Macrophage-Like Cell Lines

**Figure 4:**
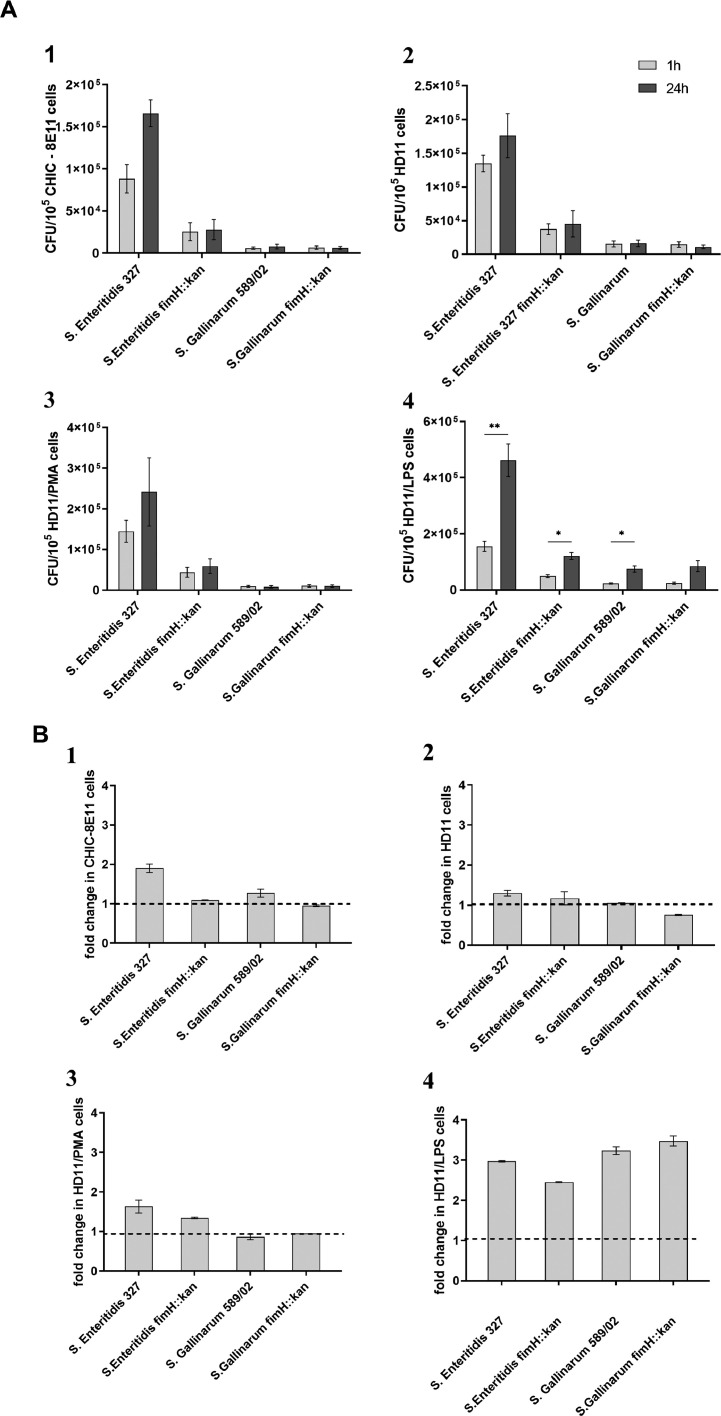
(A) Intracellular survival of wild-type *S.* Enteritidis and *S.* Gallinarum and their *S.* Enteritidis fimH::kan and *S.* Gallinarum fimH::kan mutants devoid of T1Fs in (1) chicken intestinal epithelial CHIC-8E11 cells, (2) chicken macrophage-like HD11 cells, (3) PMA- treated HD11 cells, and (4) LPS-treated HD11 cells at 1.5 h (light gray bars) and 24 h (dark gray bars) postinfection. The numbers of intracellular viable bacteria were expressed as colony forming units (CFU). (B) Fold net replication of *S.* Enteritidis, *S.* Gallinarum, *S.* Enteritidis fimH::kan and *S.* Gallinarum fimH::kan in (1) CHIC-8E11 cells, (2) HD11 cells, (3) PMA- treated HD11 cells, and (4) LPS-treated HD11 cells at 24 h postinfection. The results presented are the means ± SEM of 3 independent biological replicates, each consisting of 3 technical replicates (*n* = 9). *P < 0.05; **P < 0.01. In order to determine the statistical significance, an analysis of variance with the one-way ANOVA test was performed, and then the multiple comparison procedure was performed with the Tukey's test.

There are several lines of evidences that T1Fs play an important role in intracellular survival of *Escherichia coli*. Therefore, the role of T1Fs in the intracellular survival of *S*. Enteritidis and *S*. Gallinarum was evaluated using *S.* Enteritidis fimH::kan and *S.* Gallinarum fimH::kan mutants devoid of T1Fs. It was found that 24 h postinfection, the numbers of viable intracellular wild-type *S*. Enteritidis and *S.* Enteritidis fimH::kan strain lacking MST1Fs increased with time in CHIC-8E11 cells as well as nonactivated and PMA- or LPS-treated HD11 cells (Figure 4A). However, only in LPS-activated HD11 cells was this increase in numbers of bacteria statistically significant (Figure 4A4). In wild-type *S*. Gallinarum and its *S.* Gallinarum fimH::kan mutant, an increase in the number of intracellular bacteria was observed only for LPS-treated HD11 cells. Also, for comparison purposes, intracellular survival of bacteria was evaluated as fold change in the number of living bacteria at 24 h postinfection. No significant differences in the fold change of living bacteria inside CHIC-8E11 cells as well as nonactivated and PMA-treated HD11 cells 24 h postinfection were found between wild-type *S*. Enteritidis and *S*. Gallinarum, and their T1Fs-devoid mutant strains (Figure 4B). Again, the survival rate of all tested *Salmonella* strains was about 3-fold higher in HD11 cells treated with LPS (*P <* 0001).

### Cytotoxicity of Wild-Type *S.* Enteritidis and *S.* Gallinarum and Their T1Fs-Devoid Mutants Toward Chicken Intestinal Epithelial and Macrophage-Like Cell Lines

**Figure 5:**
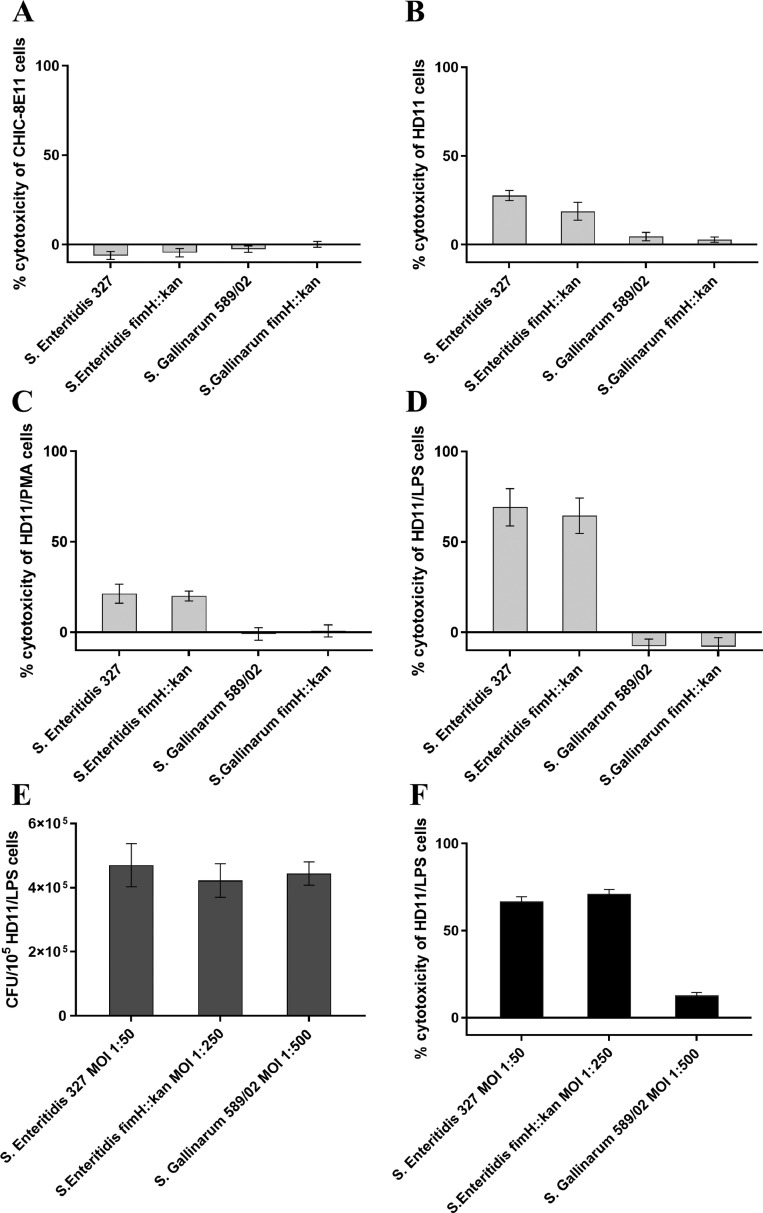
Cytotoxicity of wild-type *S.* Enteritidis and *S.* Gallinarum and their *S.* Enteritidis fimH::kan and *S.* Gallinarum fimH::kan mutants devoid of T1Fs to (A) chicken intestinal epithelial CHIC-8E11 cells, (B) chicken macrophage-like HD11 cells, (C) PMA-treated HD11 cells, (D) LPS-treated HD11 cells at 24 h postinfection. Percentage of cytotoxicity was determined by measuring the activity of lactate dehydrogenase released by infected chicken cells into culture supernatant. (E) Numbers of intracellular wild-type *S.* Enteritidis and *S.* Enteritidis fimH::kan mutant invading LPS-activated HD11 cells at an MOI, respectively, 1:50 and 1:250, and wild-type *S.* Enteritidis and wild-type *S.* Gallinarum invading LPS-activated HD11 cells at an MOI, respectively, 1:50 or 1:500 at 24 h postinfection. (F) Cytotoxicity of wild-type *S.* Enteritidis and *S.* Enteritidis fimH::kan mutant at an MOI, respectively, 1:50 or 1:250, and wild-type *S.* Enteritidis and wild-type *S.* Gallinarum to LPS-treated HD11 cells at an MOI, respectively, 1:50 or 1:500 at 24 h postinfection. The results presented are the means ± SEM of 3 independent biological replicates, each consisting of 3 technical replicates (*n* = 9).

Analysis of *Salmonella*-mediated cytotoxicity against chicken cell lines was performed by determining the activity of LDH released by infected eukaryotic cells into culture supernatant. It was found that at 24 h postinfection, wild-type *S.* Enteritidis and *S.* Gallinarum as well as their T1Fs-devoid mutants were not cytotoxic against intestinal epithelial CHIC-8E11 cells (Figure 5A). Also, *S.* Gallinarum and *S.* Gallinarum fimH::kan mutant did not show cytotoxic effects against macrophage-like HD11 cells, regardless of their activation state (Figures 5B–D). In contrast, wild-type *S*. Enteritidis and *S.* Enteritidis fimH::kan mutant were similarly cytotoxic toward nonstimulated and PMA-activated HD11 cells (% cytotoxicity—27.7% ± 9 and 21.4% ± 5.3, respectively) and similarly highly cytotoxic (69.3% ± 30.9) toward LPS-activated HD11 cells (Figures 5B–D), despite large differences in the number of invading bacteria, suggesting that the lack of MST1Fs may increase *Salmonella* cytotoxicity. Therefore, to verify this hypothesis, LPS-activated HD11 cells were infected with wild-type *S*. Enteritidis and *S.* Enteritidis fimH::kan mutant at an MOI of 1:50 and 1:250, respectively, to obtain HD11 cells with similar numbers of intracellular wild-type *S*. Enteritidis and *S.* Enteritidis fimH::kan mutant (Figure 5E). It was found that a 3.5-fold increase in the number of intracellular *S.* Enteritidis fimH::kan mutant did not increase its cytotoxicity (Figure 5F). Furthermore, to verify that differences in cytotoxicity between *S*. Enteritidis and *S*. Gallinarum were not the result of varying numbers of invading bacteria, LPS-activated HD11 cells were infected with wild-type *S*. Enteritidis and wild-type *S*. Gallinarum at an MOI of, respectively, 1:50 and 1:500 (Figure 5E). When such infected HD11 cells were subjected to cytotoxicity assay, it was found that a 6-fold increase in the number of intracellular *S*. Gallinarum did not increase its cytotoxicity (Figure 5F).

**Figure 6:**
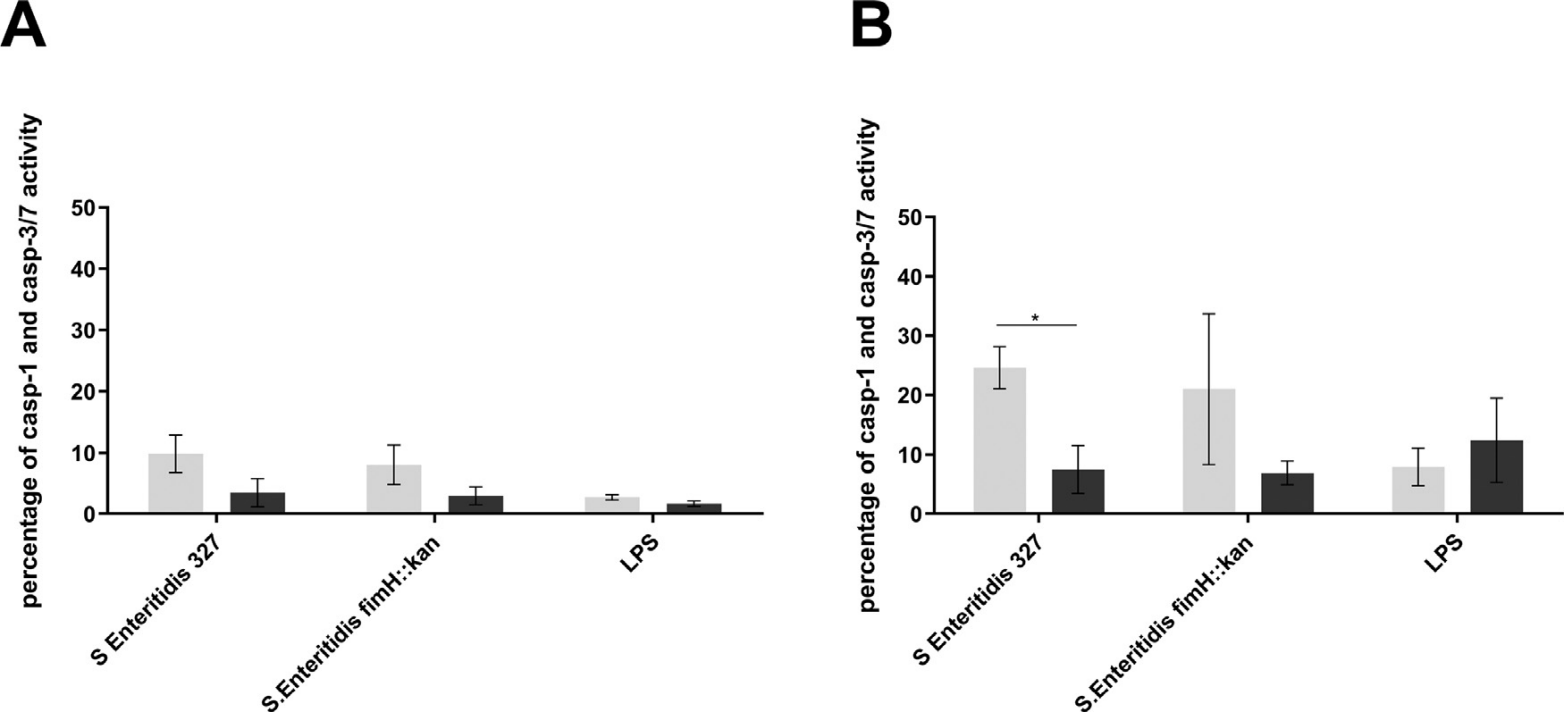
Determination of cell death type induced in LPS-treated HD11 cells by wild-type *S.* Enteritidis and *S.* Enteritidis fimH::kan mutant at 6 h (A) and 24 h (B) postinfection. The presence of an active form of caspase-1 (pyroptosis) (light gray bars) and/or active forms of caspase-3 and -7 (apoptosis) (dark gray bars) in HD11 cells were detected at 6 h and 24 h postinfection by flow cytometry using specific caspase inhibitors: Tyr-Val-Ala-Asp and Asp-Glu-Val-Asp, respectively. Flow cytometry dot plots show the percentage of pyroptotic or apoptotic cells. The results presented are the means ± SEM of 3 independent biological replicates, each consisting of 3 technical replicates (*n* = 9).

To assess the type of cell death caused by *S.* Enteritidis and its *S.* Enteritidis fimH::kan mutant, activities of apoptotic capases-3 and -7 and pyroptotic capase-1 were determined. It was found that at 6 h postinfection, 9.9% ±3.0% of LPS-treated HD11 cells were characterized by the presence of active caspase-1. At 24 h postinfection the percentage of cells with active caspase-1 increased up to 24.6% ± 3.55 in wild-type *S*. Enteritidis and 21.07 ± 12.72% in *S.* Enteritidis fimH::kan mutant (Figure 6). No LPS-activated HD11 cells with active capases-3 and -7 were found.

## DISCUSSION

The experimental data demonstrated that *S*. Enteritidis and other host unrestricted *Salmonella* serovars adhered to and invaded chicken epithelial cells or invaded and/or were taken up by chicken macrophages in significantly higher numbers than *S*. Gallinarum (Henderson et al., 1999; Jeong et al., 2008; Setta et al., 2012; Rossignol et al., 2014; Huang et al., 2019; Huang et al., 2020), however, the molecular and cellular mechanisms underlying these differences are poorly understood. It is generally accepted that effective invasion of intestinal epithelial cells by host unrestricted *Salmonella* serovars, including *S*. Enteritidis, is dependent on T3SS-1 (Haraga et al., 2008; LaRock et al., 2015; Zhang et al., 2018). In line with this view, it was shown that host-specific *S.* Gallinarum is characterized by mutations in T3SS-1 effectors (Rossignol et al., 2014; Langridge et al., 2015). Single-nucleotide polymorphism (**SNP**) found in *S*. Gallinarum *sopA, sopE*, and *sipA* genes compared to *S*. Enteritidis and *S*. Typhimurium was linked to *S*. Gallinarum low invasion of chicken and human epithelial cells. As SPI-1 genes are not required to cause fowl typhoid (Jones et al., 2001), it was proposed that other virulent factors can also participate in low or high invasive properties of these *Salmonella* serovars, including fimbriae (Huang et al., 2019). T1Fs are common and well characterized enterobacterial adhesive structures (Duguid and Gillies, 1958; Knight et al., 2000; Mulvey et al., 2000). There is evidence that mannose-sensitive (MS)-T1Fs play an important role in attachment to and colonization of gut mucosa by the majority of *Salmonella* serovars (Duguid et al., 1976; Tavendale et al., 1983; Bäumler et al., 1996). It was also found that internalization of *S*. Typhimurium by dendric cells is MST1F-dependent (Guo et al., 2007), and MST1Fs are involved in bacterial transcytosis through M cells (Hase et al., 2009). In the present study, it was confirmed that *S*. Enteritidis attached to chicken intestinal epithelial and macrophage-like cells in much higher numbers than *S*. Gallinarum, and such adhesion was MST1F-mediated in case of *S*. Enteritids. However, we were unable to show that mannose-resistant (**MR**)-T1Fs are involved in the adhesion of *S*. Gallinarum to chicken cells, and therefore we could not confirm an earlier proposal that MRT1Fs mediate the adhesion of *S*. Gallniarum to chicken leukocytes (Guo et al., 2009; Kuźmińska-Bajor et al., 2012). Importantly, adhesion positively correlated with invasion, which is in agreement with earlier studies showing that *S*. Enteritidis invaded host cells much more efficiently than *S*. Gallinarum (Rossignol et al., 2014; Kuźmińska-Bajor et al., 2015; Huang et al., 2019). Therefore, we propose that MST1Fs have a significant impact on the level of host–cell invasion by *Salmonella* as MST1F-mediated adhesion facilitates direct and stable contact of bacteria with host cells and therefore enable more efficient T3SS-1-mediated invasion. This hypothesis is supported by the following findings. The presence of MST1Fs promoted the invasion of *S*. Typhimurium and *S*. Braenderup (Ernst et al., 1990; Horiuchi et al., 1992). Mutated *S*. Typhimurium strain unable to express MST1Fs, which loss the ability to adhere to HeLa cells, was also unable to invade these cells (Bäumler et al., 1996). *S*. Gallinarum and *S*. Pullorum expressing *S*. Typhimurium MST1Fs exhibited a 10- to 20-fold increased adhesion and 20- to 60-fold increased invasion efficiency of HeLa cells (Wilson et al., 2000). However, in contrast to previous findings showing that the numbers of internalized bacteria were much higher in macrophage-like HD11 than epithelial cells (Setta et al., 2012), no differences in the numbers of internalized bacteria were found between chicken intestinal epithelial cells and chicken macrophages.

*Salmonella* as an intracellular pathogen survives inside various nonphagocytic and phagocytic host cells. It was found that despite profound differences in pathogenicity viability rate of host-unrestricted *S.* Typhimurium*, S.* Enteritidis, *S.* Hadar and *S.* Infantis and host-specific *S.* Gallinarum and *S.* Pullorum invading epithelial CKCs was essentially the same and for all analyzed serovars was 100% (Setta et al., 2012). Small differences in intracellular viability between *S*. Enteritidis and *S*. Gallinarum after invasion of chicken intestinal epithelial CHIC-8E11 cells were found in our study, since numbers of bacteria increased during 24 h postinfection in *S*. Enteritidis, and remained unchanged in *S.* Gallinarum, but for both serovars was not smaller than 100%.

Studies devoted to the intracellular survival of *Salmonella* in monocytes and macrophages brought inconclusive results. In chicken macrophage-like HD11 and MQ-NCSU cells, and chicken primary macrophages, the numbers of intracellular viable *S.* Typhimurium, *S.* Enteritidis, *S.* Hadar and *S.* Infantis as well as *S.* Gallinarum and *S*. Pullorum highly decreased after infection, indicating that survival of *Salmonella* was independent of the serovar, and was reduced over time (Kramer et al., 2003; Setta et al., 2012; Huang et al., 2019; Huang et al., 2020). However, a higher survival rate was observed for *S*. Gallinarum than for *S*. Typhimurium and *S*. Dublin, when infecting macrophages (Huang et al., 2019; Huang et al., 2020). On the other hand, total intracellular bacterial count did not change significantly or even increased postinfection in *S.* Enteritidis, *S.* Typhimurium, *S*. Infantis, *S*. Dublin and *S.* Gallinarum infecting HD11 cells or chicken peripheral blood mononuclear cell-derived (Stabler et al., 1994; Okamura et al., 2005; He et al., 2012; Blondel et al., 2013; Braukmann et al., 2015; Campioni et al., 2021). Our results obtained with macrophage-like HD11 cells revealed that the total intracellular *S*. Enteritidis and *S*. Gallinarum count also increased during 24 h postinfection. Interestingly, in LPS-activated HD11 cells, the numbers of intracellular bacteria for all analyzed *Salmonella* strains were 3-fold higher than in other chicken cells. These discrepancies may be the results of different MOI used to infect macrophages and macrophage-like cell lines and/or differences in bacterial culture conditions. In our study, *Salmonella* used to infect macrophages was grown in a stationary growth rate phase, which is optimal for T1Fs expression, in contrast to other studies, where bacteria were grown in a logarithmic growth phase optimal for T3SS-1 expression (Setta et al., 2012; Huang et al., 2019; Huang et al., 2020). Since cytotoxicity of *Salmonella* to macrophages is much higher in the transition from the exponential to the stationary phase than in stationary growth phase (Lundberg et al., 1999), the decrease in the number of viable *Salmonella* in macrophages could be the result of a decreased number of viable macrophages.

It was found that MST1Fs as well as MRT1Fs did not affected intracellular *Salmonella* viability since survival rates for, respectively, wild-type *S*. Enteritidis and *S*. Gallinarum and their T1Fs-devoid mutants were essentially the same. However, absolute numbers of intracellular viable wild-type *S.* Enteritidis were significantly higher than *S.* Enteritidis fimH::kan mutant and wild-type *S*. Gallinarum and *S.* Gallinarum fimH::kan mutant. These differences in the numbers of adherent and invading bacteria, underline the importance of MST1Fs in the pathogenicity of *Salmonella* infections.

*Salmonella* cytotoxicity to macrophages and nonphagocytic cells is a well-known phenomenon (Chen et al., 1996; Cerquetti et al., 2002). Depending on the stage of infection *Salmonella*-mediated cytotoxicity can be a beneficial for a pathogen (Lindgren et al., 1996; Monack et al., 2000) or it can be a manifestation of host defense (Fink and Cookson, 2007; Fattinger et al., 2021a,b). In this study, the cytotoxicity of host-unrestricted *S*. Enteritidis and host-specific *S*. Gallinarum to chicken intestinal epithelial cells and macrophages was compared and the role of MST1Fs and MRT1Fs in this phenomenon was evaluated. It was found that both *Salmonella* serovars are not cytotoxic to chicken intestinal epithelial CHIC-8E11 cells during 24 h postinfection, which is in contrast to other studies using various cell lines of epithelial origin (Kim et al., 1998; Paesold et al., 2002; Schauser et al., 2005; Knodler et al., 2010; Holly et al., 2020). These discrepancies, as it was discussed for *Salmonella* intracellular survival, can be the result of different *Salmonella* culture conditions used by us in comparison to other authors as it is highly accepted that the main factors responsible for *Salmonella* cytotoxicity against epithelial cells are T3SS-1 and flagella (Vance, 2015; Rauch et al., 2017; Fattinger et al., 2021a,b). In our study, *Salmonella* was cultured under conditions optimal for T1F expression, which are different from conditions that induce T3SS-1 expression.

In macrophage infection, it was found that only *S.* Enteritidis, in contrast to *S*. Gallinarum, caused considerable cell death. This is in agreement with other studies, which show that the cytotoxicity of *S.* Typhimurium, representing other host unrestricted serovars, toward HD11 cells was significantly higher than *S.* Gallinarum (Huang et al., 2019; Huang et al., 2020). However, no differences in cytotoxicity to HD11 between *S.* Enteritidis and *S.* Pullorum were found by Setta et al. (2012). We further demonstrated that high cytotoxicity of *S*. Enteritidis in comparison to *S*. Gallinarum was the intrinsic property of infecting serovar and not the result of differences in the numbers of intracellular viable bacteria, since differences in cytotoxicity still remained, when HD11 cells with essentially the same numbers of intracellular *S*. Enteritidis or *S*. Gallinarum were analyzed. It was also found that the cytotoxicity of wild-type *S.* Enteritidis and its mutant devoid of MST1Fs to HD11 cells was essentially the same, despite the fact that the number of viable intracellular bacteria was significantly lower in the mutated strain. It raises the possibility that the absence of MST1Fs may increase the cytotoxicity of *S*. Enteritidis. Using, as above, HD11 cells with essentially the same number of intracellular wild-type *S*. Enteritidis and *S.* Enteritidis fimH::kan mutant, it was found that the lack of MST1Fs did not affect directly the cytotoxicity, and the increase in cytotoxicity of mutated S. Enteritidis devoid of MST1Fs may be associated with crosstalk between T1F and other virulence factors (Kolenda et al., 2019).

Interestingly, LPS-stimulated HD11 cells were significantly more susceptible (2.5-fold) to death induction than nonstimulated and PMA-stimulated HD11 cells. As LPS-stimulated monocytes differentiate to macrophages that exhibit pro-inflammatory phenotype (Zhou et al., 2020), and monocytic cell lines, e.g. THP-1, treated with PMA differentiate to macrophages, which do not show pro-inflammatory activity, such differences can be responsible for increased sensitivity of LPS-treated cells to death in response to *Salmonella* infection. In support of this proposal, LPS is known to activate caspes-11, which leads to noncanonical inflammasome activation, and subsequently pyroptotic cell death (Shi et al., 2014; Yi, 2017; Zamyatina and Heine, 2020). Furthermore, it was shown that LPS inhibits production of nitrogen oxide in chicken peripheral blood mononuclear cell-derived macrophages (Okamura et al., 2005). As NO is one of the major oxidative burst factors during bacterial infection (He et al., 2011), it can be the reason for higher *S.* Enteritidis cytotoxicity to LPS-stimulated HD11 cells and better intracellular survival as was shown in this study. Our results indicated that cytotoxicity of *S.* Enteritidis results mainly from the activation of caspase-1, and therefore induction of the programmed death pathway defined as pyroptosis. This agrees with other studies on *S.* Typhimurium and *S.* Enteritidis and chicken and mice macrophages (Van der Velden et al., 2000; Monack et al., 2001; Thurston et al., 2016; Guo et al., 2020; Huang et al., 2022).

In summary, our study highlights the importance of MST1Fs not only during the intestinal phase, but also in infection of macrophages by *S*. Enteritidis, since the levels of invasion and survival in epithelial cells and macrophages are heavily dependent on MST1Fs-mediated adhesion. However, unlike to *E. coli*, MST1Fs are not directly involved in intracellular survival of *Salmonella*. As long as the functions of MST1Fs seem well established, the specific biological role of MRT1Fs expressed by *S*. Gallinarum remains obscure. At this point of our research, we can only speculate that the loss of binding activity by MRT1Fs may help *S.* Gallinarum to overcome the intestinal barrier and develop systemic infections in the form of fowl typhoid, as it was proposed that MST1Fs play an important role in induction of intestinal inflammation, limiting infection to the gastrointestinal tract (Kuźmińska-Bajor et al., 2015).

## Appendix Supplementary materials

Image, application 1

## References

[bib0001] Abebe E., Gugsa G., Ahmed M. (2020). Review on major food-borne zoonotic bacterial pathogens. J. Trop. Med..

[bib0002] Ali A., Kolenda R., Khan M.M., Weinreich J., Li G., Wieler L.H., Tedin K., Roggenbuck D., Schierack P. (2020). Novel avian pathogenic *Escherichia coli* genes responsible for adhesion to chicken and human cell lines. Appl. Environ. Microbiol..

[bib0003] Allen-Vercoe E., Woodward M.J. (1999). Colonisation of the chicken caecum by afimbriate and aflagellate derivatives of *Salmonella enterica* serotype Enteritidis. Vet. Microbiol..

[bib0004] Allen-Vercoe E., Woodward M.J. (1999). The role of flagella, but not fimbriae, in the adherence of *Salmonella enterica* serotype Enteritidis to chick gut explant. J. Med. Microbiol..

[bib104] Authority E.F.S. (2014). European Centre for Disease Prevention and Control. The European Union summary report on trends and sources of zoonoses, zoonotic agents and food-borne outbreaks in 2012. EFSA Journal.

[bib0005] Barrow P.A., Neto O.C.F. (2011). Pullorum disease and fowl typhoid—new thoughts on old diseases: a review. Avian Pathol..

[bib0006] Bäumler A.J., Tsolis R.M., Ficht T.A., Adams L.G. (1998). Evolution of host adaptation in *Salmonella enterica*. Infect. Immun..

[bib0007] Bäumler A.J., Tsolis R.M., Heffron F. (1996). Contribution of fimbrial operons to attachment to and invasion of epithelial cell lines by *Salmonella typhimurium*. Infect. Immun..

[bib0008] Beug H., von Kirchbach A., Döderlein G., Conscience J.-F., Graf T. (1979). Chicken hematopoietic cells transformed by seven strains of defective avian leukemia viruses display three distinct phenotypes of differentiation. Cell.

[bib0009] Blondel C.J., Jiménez J.C., Leiva L.E., Álvarez S.A., Pinto B.I., Contreras F., Pezoa D., Santiviago C.A., Contreras I. (2013). The type VI secretion system encoded in *Salmonella* pathogenicity island 19 is required for *Salmonella enterica* serotype Gallinarum survival within infected macrophages. Infect. Immun..

[bib0010] Braukmann M., Methner U., Berndt A. (2015). Immune reaction and survivability of *Salmonella typhimurium* and *Salmonella* Infantis after infection of primary avian macrophages. PLoS One.

[bib0011] Campioni F., Gomes C.N., Bergamini A.M.M., Rodrigues D.P., Tiba-Casas M.R., Falcão J.P. (2021). Comparison of cell invasion, macrophage survival and inflammatory cytokines profiles between *Salmonella enterica* serovars Enteritidis and Dublin from Brazil. J. Appl. Microbiol..

[bib0012] Cerquetti M.C., Goren N.B., Ropolo A.J., Grasso D., Giacomodonato M.N., Vaccaro M.I. (2002). Nitric oxide and apoptosis induced in Peyer's patches by attenuated strains of *Salmonella enterica* serovar Enteritidis. Infect. Immun..

[bib0013] Chadfield M.S., Brown D.J., Aabo S., Christensen J.P., Olsen J.E. (2003). Comparison of intestinal invasion and macrophage response of *Salmonella* Gallinarum and other host-adapted *Salmonella enterica* serovars in the avian host. Vet. Microbiol..

[bib0014] Chen L.M., Kaniga K., Galán J.E. (1996). *Salmonella* spp. are cytotoxic for cultured macrophages. Mol. Microbiol..

[bib0015] Chlebicz A., Śliżewska K. (2018). Campylobacteriosis, salmonellosis, yersiniosis, and listeriosis as zoonotic foodborne diseases: a review. Int. J. Environ. Res. Public Health.

[bib0016] Ciesielska A., Matyjek M., Kwiatkowska K. (2021). TLR4 and CD14 trafficking and its influence on LPS-induced pro-inflammatory signaling. Cell. Mol. Life Sci..

[bib0017] Craven S.E., Cox N.A., Bailey J.S., Blankenship L.C. (1992). Binding of *Salmonella* strains to immobilized intestinal mucosal preparations from broiler chickens. Avian Dis..

[bib102] Datsenko K.A., Wanner B.L. (2000). One-step inactivation of chromosomal genes in Escherichia coli K-12 using PCR products. Proc. Natl. Acad. Sci. U.S.A..

[bib0018] De Buck J., Van Immerseel F., Haesebrouck F., Ducatelle R. (2004). Effect of type 1 fimbriae of *Salmonella enterica* serotype Enteritidis on bacteraemia and reproductive tract infection in laying hens. Avian Pathol..

[bib0019] De Buck J., Van Immerseel F., Meulemans G., Haesebrouck F., Ducatelle R. (2003). Adhesion of *Salmonella enterica* serotype Enteritidis isolates to chicken isthmal glandular secretions. Vet. Microbiol..

[bib0020] Dibb-Fuller M.P., Woodward M.J. (2000). Contribution of fimbriae and flagella of *Salmonella enteritidis* to colonization and invasion of chicks. Avian Pathol..

[bib0021] Duguid J.P., Darekar M.R., Wheater D.W. (1976). Fimbriae and infectivity in *Salmonella typhimurium*. J. Med. Microbiol..

[bib0022] Duguid J.P., Gillies R.R. (1958). Fimbriae and haemagglutinating activity in *Salmonella, Klebsiella, Proteus* and *Chromobacterium*. J. Pathol. Bacteriol..

[bib0023] Ernst R.K., Dombroski D.M., Merrick J.M. (1990). Anaerobiosis, type 1 fimbriae, and growth phase are factors that affect invasion of HEp-2 cells by *Salmonella typhimurium*. Infect. Immun..

[bib105] European Food Safety Authority and European Centre for Disease. Prevention and control (EFSA and ECDC) (2018). The European Union summary report on trends and sources of zoonoses, zoonotic agents and food-borne outbreaks in 2017. EFSA Journal.

[bib0024] European Food Safety Authority, European Centre for Disease Prevention and Control (2021).

[bib0025] European Food Safety Authority, European Centre for Disease Prevention and Control (2021).

[bib0026] Fàbrega A., Vila J. (2013). *Salmonella enterica* serovar Typhimurium skills to succeed in the host: virulence and regulation. Clin. Microbiol. Rev..

[bib0027] Fattinger S.A., Geiser P., Samperio Ventayol P., Di Martino M.L., Furter M., Felmy B., Bakkeren E., Hausmann A., Barthel-Scherrer M., Gül E., Hardt W.-D., Sellin M.E. (2021). Epithelium-autonomous NAIP/NLRC4 prevents TNF-driven inflammatory destruction of the gut epithelial barrier in *Salmonella*-infected mice. Mucosal. Immunol..

[bib0028] Fattinger S.A., Sellin M.E., Hardt W.-D. (2021). Epithelial inflammasomes in the defense against *Salmonella* gut infection. Curr. Opin. Microbiol..

[bib0029] Fields P.I., Swanson R.V., Haidaris C.G., Heffron F. (1986). Mutants of *Salmonella typhimurium* that cannot survive within the macrophage are avirulent. Proc. Natl. Acad. Sci. U.S.A..

[bib0030] Fink S.L., Cookson B.T. (2007). Pyroptosis and host cell death responses during *Salmonella* infection. Cell. Microbiol..

[bib0031] Genin M., Clement F., Fattaccioli A., Raes M., Michiels C. (2015). M1 and M2 macrophages derived from THP-1 cells differentially modulate the response of cancer cells to etoposide. BMC Cancer.

[bib0032] Guo A., Cao S., Tu L., Chen P., Zhang C., Jia A., Yang W., Liu Z., Chen H., Schifferli D.M. (2009). FimH alleles direct preferential binding of *Salmonella* to distinct mammalian cells or to avian cells. Microbiology.

[bib0033] Guo A., Lasaro M.A., Sirard J.-C., Kraehenbühl J.-P., Schifferli D.M. (2007). Adhesin-dependent binding and uptake of *Salmonella enterica* serovar Typhimurium by dendritic cells. Microbiology (Reading).

[bib0034] Guo Y., Gu D., Huang T., Cao L., Zhu X., Zhou Y., Wang K., Kang X., Meng C., Jiao X., Pan Z. (2020). Essential role of *Salmonella* Enteritidis DNA adenine methylase in modulating inflammasome activation. BMC Microbiol..

[bib0035] Haraga A., Ohlson M.B., Miller S.I. (2008). Salmonellae interplay with host cells. Nat. Rev. Microbiol..

[bib0036] Hase K., Kawano K., Nochi T., Pontes G.S., Fukuda S., Ebisawa M., Kadokura K., Tobe T., Fujimura Y., Kawano S., Yabashi A., Waguri S., Nakato G., Kimura S., Murakami T., Iimura M., Hamura K., Fukuoka S.-I., Lowe A.W., Itoh K., Kiyono H., Ohno H. (2009). Uptake through glycoprotein 2 of FimH(+) bacteria by M cells initiates mucosal immune response. Nature.

[bib0037] He H., Genovese K.J., Kogut M.H. (2011). Modulation of chicken macrophage effector function by TH1/TH2 cytokines. Cytokine.

[bib0038] He H., Genovese K.J., Swaggerty C.L., Nisbet D.J., Kogut M.H. (2012). A comparative study on invasion, survival, modulation of oxidative burst, and nitric oxide responses of macrophages (HD11), and systemic infection in chickens by prevalent poultry *Salmonella* serovars. Foodborne Pathog. Dis..

[bib0039] Henderson S.C., Bounous D.I., Lee M.D. (1999). Early events in the pathogenesis of avian salmonellosis. Infect. Immun..

[bib0040] Holly M.K., Han X., Zhao E.J., Crowley S.M., Allaire J.M., Knodler L.A., Vallance B.A., Smith J.G. (2020). *Salmonella enterica* infection of murine and human enteroid-derived monolayers elicits differential activation of epithelium-intrinsic inflammasomes. Infect. Immun..

[bib0041] Horiuchi S., Inagaki Y., Okamura N., Nakaya R., Yamamoto N. (1992). Type 1 pili enhance the invasion of *Salmonella* braenderup and *Salmonella typhimurium* to HeLa cells. Microbiol. Immunol..

[bib0042] Huang K., Fresno A.H., Skov S., Olsen J.E. (2020). Dynamics and outcome of Macrophage Interaction between Salmonella Gallinarum, Salmonella Typhimurium, and Salmonella Dublin and Macrophages from chicken and cattle. Front. Cell. Infect. Microbiol..

[bib0043] Huang K., Herrero-Fresno A., Thøfner I., Skov S., Olsen J.E. (2019). Interaction differences of the avian host-specific *Salmonella enterica* serovar Gallinarum, the host-generalist *S.* Typhimurium, and the cattle host-adapted *S.* Dublin with chicken primary macrophage. Infect. Immun..

[bib0044] Huang T., Gu D., Guo Y., Li A., Kang X., Jiao X., Pan Z. (2022). *Salmonella* Enteritidis GalE protein inhibits LPS-induced NLRP3 inflammasome activation. Microorganisms.

[bib0045] Jeong J.-H., Song M., Park S.-I., Cho K.-O., Rhee J.H., Choy H.E. (2008). *Salmonella enterica* serovar Gallinarum requires ppGpp for internalization and survival in animal cells. J. Bacteriol..

[bib0046] Jones M.A., Wigley P., Page K.L., Hulme S.D., Barrow P.A. (2001). *Salmonella enterica* serovar Gallinarum requires the *Salmonella* pathogenicity island 2 type III secretion system but not the *Salmonella* pathogenicity island 1 type III secretion system for virulence in chickens. Infect. Immun..

[bib0047] Kim J.M., Eckmann L., Savidge T.C., Lowe D.C., Witthöft T., Kagnoff M.F. (1998). Apoptosis of human intestinal epithelial cells after bacterial invasion. J. Clin. Invest..

[bib0048] Kisiela D., Laskowska A., Sapeta A., Kuczkowski M., Wieliczko A., Ugorski M. (2006). Functional characterization of the FimH adhesin from Salmonella enterica serovar enteritidis. Microbiology.

[bib0049] Kisiela D., Sapeta A., Kuczkowski M., Stefaniak T., Wieliczko A., Ugorski M. (2005). Characterization of FimH adhesins expressed by *Salmonella enterica* serovar Gallinarum biovars Gallinarum and Pullorum: reconstitution of mannose-binding properties by single amino acid substitution. Infect. Immun..

[bib0050] Knight S., Berglund J., Choudhury D. (2000). Bacterial adhesins: structural studies reveal chaperone function and pilus biogenesis. Curr. Opin. Chem. Biol..

[bib0051] Knodler L.A., Vallance B.A., Celli J., Winfree S., Hansen B., Montero M., Steele-Mortimer O. (2010). Dissemination of invasive *Salmonella* via bacterial-induced extrusion of mucosal epithelia. Proc. Natl. Acad. Sci. U. S. A..

[bib0052] Kogut M.H., Tellez G.I., McGruder E.D., Hargis B.M., Williams J.D., Corrier D.E., DeLoach J.R. (1994). Heterophils are decisive components in the early responses of chickens to *Salmonella* enteritidis infections. Microbial. Pathog..

[bib0053] Kolenda R., Ugorski M., Grzymajlo K. (2019). Everything you always wanted to know about *Salmonella* type 1 fimbriae, but were afraid to ask. Front. Microbiol..

[bib0054] Kramer J., Visscher A.H., Wagenaar J.A., Jeurissen S.H.M. (2003). Entry and survival of *Salmonella enterica* serotype Enteritidis PT4 in chicken macrophage and lymphocyte cell lines. Vet. Microbiol..

[bib0055] Kuźmińska-Bajor M., Grzymajło K., Ugorski M. (2015). Type 1 fimbriae are important factors limiting the dissemination and colonization of mice by *Salmonella* Enteritidis and contribute to the induction of intestinal inflammation during *Salmonella* invasion. Front. Microbiol..

[bib0056] Kuźmińska-Bajor M., Kuczkowski M., Grzymajło K., Wojciech Ł., Sabat M., Kisiela D., Wieliczko A., Ugorski M. (2012). Decreased colonization of chicks by *Salmonella enterica* serovar Gallinarum expressing mannose-sensitive FimH adhesin from *Salmonella enterica* serovar Enteritidis. Vet. Microbiol..

[bib0058] Langridge G.C., Fookes M., Connor T.R., Feltwell T., Feasey N., Parsons B.N., Seth-Smith H.M.B., Barquist L., Stedman A., Humphrey T., Wigley P., Peters S.E., Maskell D.J., Corander J., Chabalgoity J.A., Barrow P., Parkhill J., Dougan G., Thomson N.R. (2015). Patterns of genome evolution that have accompanied host adaptation in *Salmonella*. Proc. Natl. Acad. Sci..

[bib0059] LaRock D.L., Chaudhary A., Miller S.I. (2015). Salmonellae interactions with host processes. Nat. Rev. Microbiol..

[bib0060] Lindgren S.W., Stojiljkovic I., Heffron F. (1996). Macrophage killing is an essential virulence mechanism of *Salmonella typhimurium*. Proc. Natl. Acad. Sci. U. S. A..

[bib0061] Lund M.E., To J., O'Brien B.A., Donnelly S. (2016). The choice of phorbol 12-myristate 13-acetate differentiation protocol influences the response of THP-1 macrophages to a pro-inflammatory stimulus. J. Immunol. Methods.

[bib0062] Lundberg U., Vinatzer U., Berdnik D., von Gabain A., Baccarini M. (1999). Growth phase-regulated induction of *Salmonella*-induced macrophage apoptosis correlates with transient expression of SPI-1 genes. J. Bacteriol..

[bib0063] Milczarek M., Sadkowska-Todys M., Czarkowski M.P., Kitowska W. (2019). Salmonellosis in Poland in 2017. Przegl. Epidemiol..

[bib0064] Monack D.M., Detweiler C.S., Falkow S. (2001). Salmonella pathogenicity island 2-dependent macrophage death is mediated in part by the host cysteine protease caspase-1. Cell. Microbiol..

[bib0065] Monack D.M., Hersh D., Ghori N., Bouley D., Zychlinsky A., Falkow S. (2000). *Salmonella* exploits caspase-1 to colonize Peyer's patches in a murine typhoid model. J. Exp. Med..

[bib0066] Mulvey M.A., Schilling J.D., Martinez J.J., Hultgren S.J. (2000). Bad bugs and beleaguered bladders: interplay between uropathogenic *Escherichia coli* and innate host defenses. Proc. Natl. Acad. Sci. U. S. A..

[bib0067] Okamura M., Lillehoj H.S., Raybourne R.B., Babu U.S., Heckert R.A., Tani H., Sasai K., Baba E., Lillehoj E.P. (2005). Differential responses of macrophages to *Salmonella enterica* serovars Enteritidis and Typhimurium. Vet. Immunol. Immunopathol..

[bib0068] Oyofo B.A., Droleskey R.E., Norman J.O., Mollenhauer H.H., Ziprin R.L., Corrier D.E., DeLoach J.R. (1989). Inhibition by mannose of in vitro colonization of chicken small intestine by *Salmonella typhimurium*. Poult. Sci..

[bib0069] Paesold G., Guiney D.G., Eckmann L., Kagnoff M.F. (2002). Genes in the *Salmonella* pathogenicity island 2 and the *Salmonella* virulence plasmid are essential for *Salmonella*-induced apoptosis in intestinal epithelial cells. Cell. Microbiol..

[bib0070] Plym F.L., Wierup M. (2006). Salmonella contamination: a significant challenge to the global marketing of animal food products. Rev. Sci. Tech..

[bib0071] Rajashekara G., Munir S., Alexeyev M.F., Halvorson D.A., Wells C.L., Nagaraja K.V. (2000). Pathogenic role of SEF14, SEF17, and SEF21 fimbriae in *Salmonella enterica* serovar Enteritidis infection of chickens. Appl. Environ. Microbiol..

[bib0072] Rauch I., Deets K.A., Ji D.X., von Moltke J., Tenthorey J.L., Lee A.Y., Philip N.H., Ayres J.S., Brodsky I.E., Gronert K., Vance R.E. (2017). NAIP-NLRC4 inflammasomes coordinate intestinal epithelial cell expulsion with eicosanoid and IL-18 release via activation of caspase-1 and -8. Immunity.

[bib0073] Rossignol A., Roche S.M., Virlogeux-Payant I., Wiedemann A., Grépinet O., Fredlund J., Trotereau J., Marchès O., Quéré P., Enninga J., Velge P. (2014). Deciphering why *Salmonella* Gallinarum is less invasive in vitro than *Salmonella* Enteritidis. Vet. Res..

[bib0074] Schauser K., Olsen J.E., Larsson L.-I. (2005). *Salmonella typhimurium* infection in the porcine intestine: evidence for caspase-3-dependent and -independent programmed cell death. Histochem. Cell. Biol..

[bib0075] Setta A., Barrow P.A., Kaiser P., Jones M.A. (2012). Immune dynamics following infection of avian macrophages and epithelial cells with typhoidal and non-typhoidal *Salmonella enterica* serovars; bacterial invasion and persistence, nitric oxide and oxygen production, differential host gene expression, NF-κB signalling and cell cytotoxicity. Vet. Immunol. Immunopathol..

[bib0076] Shi J., Zhao Y., Wang Y., Gao W., Ding J., Li P., Hu L., Shao F. (2014). Inflammatory caspases are innate immune receptors for intracellular LPS. Nature.

[bib0077] Shivaprasad H.L. (2000). Fowl typhoid and pullorum disease: -EN- -FR- -ES-. Rev. Sci. Tech..

[bib0078] Stabler J.G., McCormick T.W., Powell K.C., Kogut M.H. (1994). Avian heterophils and monocytes: phagocytic and bactericidal activities against *Salmonella* enteritidis. Vet. Microbiol..

[bib0079] Strober W. (2001). Trypan blue exclusion test of cell viability. Curr. Protoc. Immunol..

[bib0080] Sun F., Li X., Wang Y., Wang F., Ge H., Pan Z., Xu Y., Wang Y., Jiao X., Chen X. (2021). Epidemic patterns of antimicrobial resistance of *Salmonella enterica* serovar Gallinarum biovar Pullorum isolates in China during the past half-century. Poult. Sci..

[bib0081] Tavendale A., Jardine C.K., Old D.C., Duguid J.P. (1983). Haemagglutinins and adhesion of *Salmonella typhimurium* to HEp2 and HeLa cells. J. Med. Microbiol..

[bib0084] Thiagarajan D., Thacker H.L., Saeed A.M. (1996). Experimental infection of laying hens with *Salmonella* Enteritidis strains that express different types of fimbriae. Poult. Sci..

[bib0085] Thomson N.R., Clayton D.J., Windhorst D., Vernikos G., Davidson S., Churcher C., Quail M.A., Stevens M., Jones M.A., Watson M., Barron A., Layton A., Pickard D., Kingsley R.A., Bignell A., Clark L., Harris B., Ormond D., Abdellah Z., Brooks K., Cherevach I., Chillingworth T., Woodward J., Norberczak H., Lord A., Arrowsmith C., Jagels K., Moule S., Mungall K., Sanders M., Whitehead S., Chabalgoity J.A., Maskell D., Humphrey T., Roberts M., Barrow P.A., Dougan G., Parkhill J. (2008). Comparative genome analysis of *Salmonella* Enteritidis PT4 and Salmonella Gallinarum 287/91 provides insights into evolutionary and host adaptation pathways. Genome Res..

[bib0086] Thurston T.L.M., Matthews S.A., Jennings E., Alix E., Shao F., Shenoy A.R., Birrell M.A., Holden D.W. (2016). Growth inhibition of cytosolic *Salmonella* by caspase-1 and caspase-11 precedes host cell death. Nat. Commun..

[bib0087] Ugorski M., Kuzminska-Bajor M., Kisiela D. (2011). Rola fimbrii typu 1 w patogenezie zakażeń pałeczkami *Salmonella*. Postępy Mikrobiologii..

[bib0088] Uzzau S., Brown D.J., Wallis T., Rubino S., Leori G., Bernard S., Casadesús J., Platt D.J., Olsen J.E. (2000). Host adapted serotypes of *Salmonella enterica*. Epidemiol. Infect..

[bib0089] Vaid R.K., Thakur Z., Anand T., Kumar S., Tripathi B.N. (2021). Comparative genome analysis of *Salmonella enterica* serovar Gallinarum biovars Pullorum and Gallinarum decodes strain specific genes. PLoS One.

[bib0090] Vance R.E. (2015). The NAIP/NLRC4 Inflammasomes. Curr. Opin. Immunol..

[bib0091] van der Velden A.W., Lindgren S.W., Worley M.J., Heffron F. (2000). Salmonella pathogenicity island 1-independent induction of apoptosis in infected macrophages by *Salmonella enterica* serotype typhimurium. Infect. Immun..

[bib0092] Vazquez-Torres A., Jones-Carson J., Bäumler A.J., Falkow S., Valdivia R., Brown W., Le M., Berggren R., Parks W.T., Fang F.C. (1999). Extraintestinal dissemination of *Salmonella* by CD18-expressing phagocytes. Nature.

[bib0093] Wigley P., Jones M.A., Barrow P.A. (2002). *Salmonella enterica* serovar Pullorum requires the *Salmonella* pathogenicity island 2 type III secretion system for virulence and carriage in the chicken. Avian Pathol..

[bib0094] Wilson R.L., Elthon J., Clegg S., Jones B.D. (2000). *Salmonella enterica* serovars Gallinarum and Pullorum expressing *Salmonella enterica* serovar Typhimurium type 1 fimbriae exhibit increased invasiveness for mammalian cells. Infect. Immun..

[bib0095] Yi Y.-S. (2017). Caspase-11 non-canonical inflammasome: a critical sensor of intracellular lipopolysaccharide in macrophage-mediated inflammatory responses. Immunology.

[bib0096] Zamyatina A., Heine H. (2020). Lipopolysaccharide recognition in the crossroads of TLR4 and caspase-4/11 mediated inflammatory pathways. Front. Immunol..

[bib0097] Zeiner S.A., Dwyer B.E., Clegg S. (2012). FimA, FimF, and FimH are necessary for assembly of type 1 fimbriae on *Salmonella enterica* serovar Typhimurium. Infect. Immun..

[bib0098] Zhang J.-F., Shang K., Park J.-Y., Lee Y.-J., Choi Y.-R., Kim S.-W., Cha S.-Y., Jang H.-K., Wei B., Kang M. (2021). Antimicrobial resistance and PFGE molecular typing of *Salmonella enterica* serovar Gallinarum isolates from chickens in South Korea from 2013 to 2018. Animals (Basel).

[bib0099] Zhang K., Riba A., Nietschke M., Torow N., Repnik U., Pütz A., Fulde M., Dupont A., Hensel M., Hornef M. (2018). Minimal SPI1-T3SS effector requirement for Salmonella enterocyte invasion and intracellular proliferation in vivo. PLoS Pathog..

[bib0100] Zhou P., Li Q., Su S., Dong W., Zong S., Ma Q., Yang X., Zuo D., Zheng S., Meng X., Xu D., Zeng Q. (2020). Interleukin 37 suppresses M1 macrophage polarization through inhibition of the Notch1 and nuclear factor kappa B pathways. Front. Cell Dev. Biol..

