# Supplementary material for: Unraveling the role of type 1 fimbriae in Salmonella pathogenesis: insights from a comparative analysis of Salmonella Enteritidis and Salmonella Gallinarum
Source: Poult Sci. 2023 Jun 3;102(8):102833. doi: 10.1016/j.psj.2023.102833 (PMC10404763; doi:10.1016/j.psj.2023.102833)

**Figure S.1**

Dot plot of flow cytometry analysis determining the type of cell death induced in LPS-treated HD11 cells as a result of *S.* Enteritidis 327 and S.Enteritidis fimH:: kan infection with the *fimH* gene deletion. The presence of an active form of caspase 1 (pyroptosis) (A1,B1) and / or active forms of caspase 3 and 7 (apoptosis) (A2,B2) was detected in HD11 cells at 6 hours (A) and 24 hours (B) after infection using specific tetrapeptide caspase inhibitors Tyr-Val-Ala-Asp (YVAD) and Asp-Glu-Val-Asp (DEVD), respectively, conjugated with APC and a commercial fluorescent dye - NucGreen.


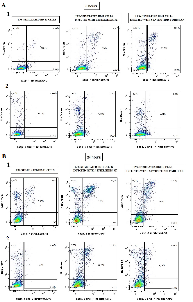

Supplement: Supplementary file 1 [file mmc1.docx]
